# Supplementary figures and images for: An Inertial Sensor-Based Gait Analysis Pipeline for the Assessment of Real-World Stair Ambulation Parameters
Source: Sensors (Basel). 2021 Sep 30;21(19):6559. doi: 10.3390/s21196559 (PMC8513040; doi:10.3390/s21196559)

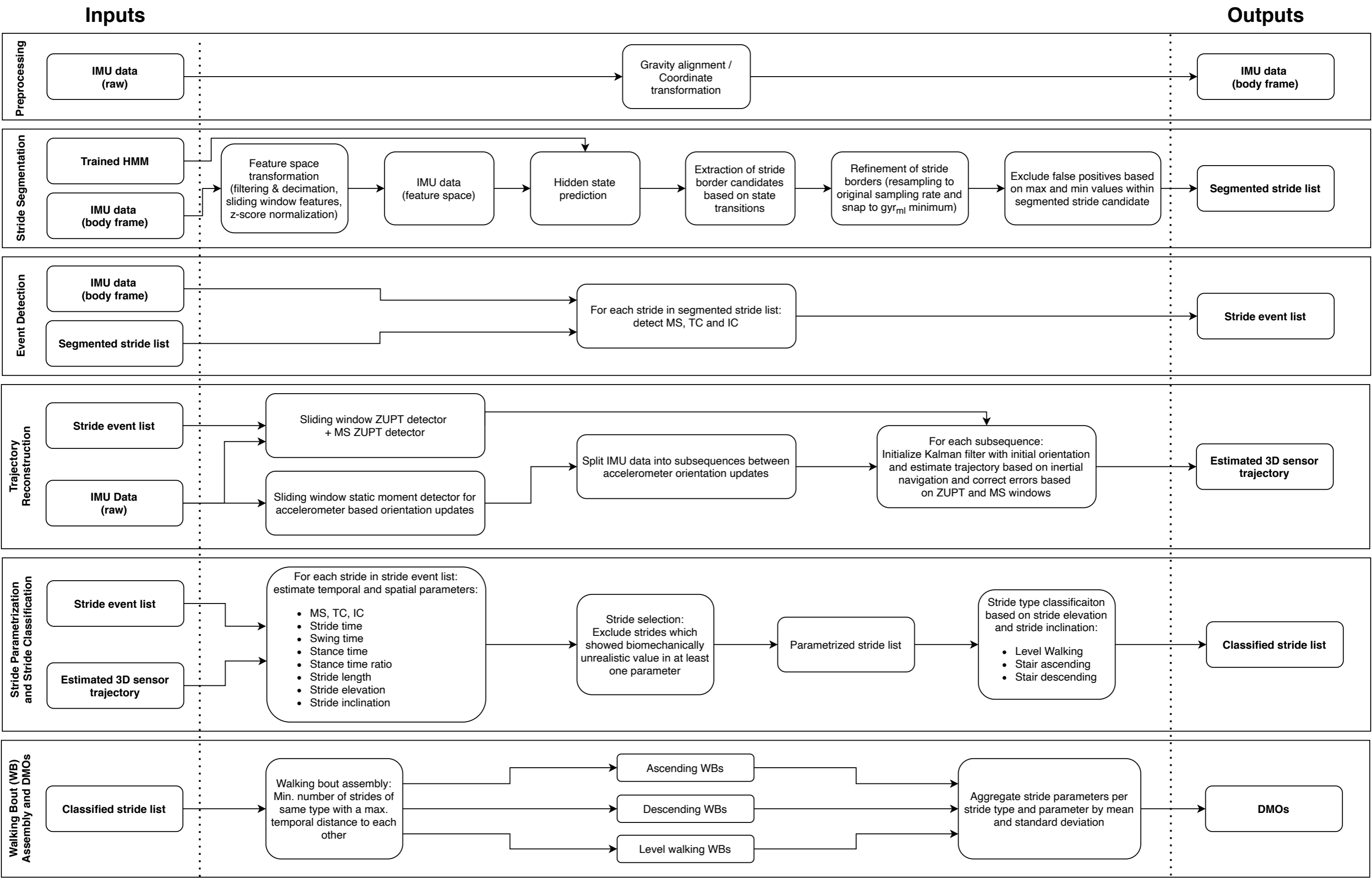

Supplement: Supplementary file 1 [file sensors-21-06559-s001.zip › sensors-1405137 (supplementary).pdf]
